# The COVID-19 Pandemic Is Over, but the Virus Still Lingers

**DOI:** 10.3390/diseases12030057

**Published:** 2024-03-19

**Authors:** Ludovico Abenavoli

**Affiliations:** Department of Health Sciences, University “Magna Graecia”, Viale Europa, 88100 Catanzaro, Italy; l.abenavoli@unicz.it; Tel.: +39-0961-3694387

The global health emergency caused by the Coronavirus disease-19 (COVID-19) pandemic officially ended on 11 May 2023. However, COVID-19 is unfortunately still present; its devastating effects have been largely overcome, but they have left an unforgettable impression on us all. The COVID-19 pandemic resulted in human stress with serious and long-term implications for the health, quality of life, and psychological wellness of people worldwide [1]. On the other side, the COVID-19 pandemic provided an unexpected window of opportunity to see things in a way we had not thought of before.

The pandemic has left us with written documents, studies, and evidence of the immense work that doctors, scholars, and all researchers across the globe have carried out to initially understand “what” Severe Acute Respiratory Syndrome Coronavirus-2 was, to define its diagnosis, to find treatments, to discover new vaccines against COVID-19, and finally to provide adequate health systems to meet this global challenge. This was the first global pandemic that the scientific publishing industry had ever faced, and the first of the digital era of communication and internet publishing. During the emergency phase, studies were focused on the field of effectiveness of vaccines and new therapies; subsequently, scholars focused their attention on understanding what the predisposing factors for infections and more serious outcomes are, in addition to previous diseases, in the clinical scenarios of “post-acute sequelae of COVID-19” and/or “long COVID” [2].

In fact, many important advances have been achieved in the domain of COVID-19, and in particular: (1) the definition of molecular mechanisms that regulate Coronavirus pathogenesis; (2) the use of effective vaccines in real-world settings, especially when the Omicron variant was dominant, with a reduction in mortality; (3) the development of antiviral drugs with additional protection for subjects at higher risk of severe disease; (4) the upgrade of health system organization in several countries; (5) the advances in artificial intelligence, robotics, and automation [3]. These measures are pivotal in the post-pandemic era, as COVID-19 has now transformed into a persistent but less-deadly presence [4,5].

In conclusion, the COVID-19 pandemic has opened new challenges, but it has also forced humanity to reconsider the impact of health on the quality of life. This particular period of our history has completely changed how we deliver healthcare and the way in which it is possible to conduct research. However, along with the suffering and great challenges, we have the opportunity to make a positive impact on the progress of our society. If we manage to learn from this crisis and from the approach we have used to handle it, many things will change for the better, such as the delivery of high-quality care and the analysis of more accurate data. However, without a global sanitary vision and an adequate scientific policy, both supported by adequate funds, following such a strategy is not possible.

## References

[1] DeSalvo K., Hughes B., Bassett M., Benjamin G., Fraser M., Galea S., Gracia J.N. (2021). Public Health COVID-19 Impact Assessment: Lessons Learned and Compelling Needs. NAM Perspect..

[2] Sabitha S., Shobana N., Prakash P., Padmanaban S., Sathiyashree M., Saigeetha S., Chakravarthi S., Uthaman S., Park I.-K., Samrot A.V. (2022). A Review of Different Vaccines and Strategies to Combat COVID-19. Vaccines.

[3] Fang Y., Xing X., Wang S., Walsh S., Yang G. (2024). Post-COVID highlights: Challenges and solutions of artificial intelligence techniques for swift identification of COVID-19. Curr. Opin. Struct. Biol.

[4] Mumtaz H., Riaz M.H., Wajid H., Saqib M., Zeeshan M.H., Khan S.E., Chauhan Y.R., Sohail H., Vohra L.I. (2023). Current challenges and potential solutions to the use of digital health technologies in evidence generation: A narrative review. Front. Digit. Health.

[5] Branda F., Abenavoli L., Pierini M., Mazzoli S. (2022). Predicting the Spread of SARS-CoV-2 in Italian Regions: The Calabria Case Study, February 2020–March 2022. Diseases.

